# Age-Dependent Protein Expression of Serine/Threonine Phosphatases and Their Inhibitors in the Human Cardiac Atrium

**DOI:** 10.1155/2019/2675972

**Published:** 2019-01-02

**Authors:** Ulrich Gergs, Theresa Trapp, Hasan Bushnaq, Andreas Simm, Rolf-Edgar Silber, Joachim Neumann

**Affiliations:** ^1^Institut für Pharmakologie und Toxikologie, Medizinische Fakultät, Martin-Luther-Universität Halle-Wittenberg, 06112 Halle (Saale), Germany; ^2^Klinik für Herz- und Thoraxchirurgie, Medizinische Fakultät, Martin-Luther-Universität Halle-Wittenberg, 06097 Halle, Germany

## Abstract

Heart failure and aging of the heart show many similarities regarding hemodynamic and biochemical parameters. There is evidence that heart failure in experimental animals and humans is accompanied and possibly exacerbated by increased activity of protein phosphatase (PP) 1 and/or 2A. Here, we wanted to study the age-dependent protein expression of major members of the protein phosphatase family in human hearts. Right atrial samples were obtained during bypass surgery. Patients (*n*=60) were suffering from chronic coronary artery disease (CCS 2-3; New York Heart Association (NYHA) stage 1–3). Age ranged from 48 to 84 years (median 69). All patients included in the study were given *β*-adrenoceptor blockers. Other medications included angiotensin-converting enzyme (ACE) or angiotensin-receptor-1 (AT_1_) inhibitors, statins, nitrates, and acetylsalicylic acid (ASS). 100 *µ*g of right atrial homogenates was used for western blotting. Antibodies against catalytic subunits (and their major regulatory proteins) of all presently known cardiac serine/threonine phosphatases were used for antigen detection. In detail, we studied the expression of the catalytic subunit of PP1 (PP1c); I_1_^PP1^ and I_2_^PP1^, proteins that can inhibit the activity of PP1c; the catalytic subunit of PP2A (PP2Ac); regulatory A-subunit of PP2A (PP2A_A_); regulatory B56*α*-subunit of PP2A (PP2A_B_); I_1_^PP2A^ and I_2_^PP2A^, inhibitory subunits of PP2A; catalytic and regulatory subunits of calcineurin: PP2B_A_ and PP2B_B_; PP2C; PP5; and PP6. All data were obtained within the linear range of the assay. There was a significant decline in PP2Ac and I_2_^PP2A^ expression in older patients, whereas all other parameters remained unchanged with age. It remains to be elucidated whether the decrease in the protein expression of I_2_^PP2A^ might elevate cardiac PP2A activity in a detrimental way or is overcome by a reduced protein expression and thus a reduced activity of PP2Ac.

## 1. Introduction

In the myocardium, Ca^2+^-induced Ca^2+^ release from the sarcoplasmic reticulum (SR) via activation of ryanodine receptors is the main mechanism of cardiac excitation-contraction coupling [1]. The ensuing increase in intracellular Ca^2+^ concentration is responsible for muscle contraction [1]. For relaxation, Ca^2+^ is mainly removed from the cytosol by the action of SR Ca^2+^-ATPase (SERCA) into the SR. The affinity of SERCA for Ca^2+^ is regulated by phospholamban (PLB) located in the SR. Phospholamban itself can be dephosphorylated by two serine/threonine phosphatases, namely, PP1 and PP2A in animal hearts and the human heart [2–5]. The catalytic subunit of PP1 can be inhibited by, amongst others, two endogenous proteins (for review, see [4], http://www.Phosphatome.net, and http://www.depod.org) with peculiar physicochemical properties (preserved action after boiling of samples). These heat stable proteins have been named inhibitor 1 of PP1 (I_1_^PP1^ [6]) and inhibitor 2 of PP1 (I_2_^PP1^ [6]). Long-term cardiac specific overexpression of I_1_ leads upon aging in mouse hearts to decreased systolic contractility, suggesting that low PP1 activity is also detrimental for cardiac function in the long run [7]. On the other hand, increased PP1 overexpression in the mouse heart can lead to cardiac hypertrophy and death [8]. In contrast to PP1, another related phosphatase, namely, PP2A, probably mainly exists as a trimer comprising the catalytic subunit, the B subunit, and the A subunit (for current review, see [9]). Cardiac overexpression of PP2A leads in transgenic mice to cardiac hypertrophy [10]. Interestingly, similar to PP1, additional proteins that inhibit PP2A activity have been identified, namely, inhibitor 1 of PP2A (I_1_^PP2A^ [11]) and inhibitor 2 of PP2A (I_2_^PP2A^ [12, 13]). Furthermore, a plethora of papers have described and characterized PP2B, consisting of two subunits, termed A subunit and B subunit. The A subunit binds calmodulin and contains the catalytic activity. The B subunit is a Ca^2+^-binding protein (for review, see [4]). The classical substrate for PP2B in the heart is NFAT which is involved in gene transcription. Much less is known about PP2C in the heart. We have recently generated mice with PP2C overexpression in the heart which led to mild hypertrophy upon aging [14]. It is usually assumed that PP2C is localized in the mitochondria of cardiac cells, and due to this localization it might play a role in ischemia [4]. Finally, PP5 and PP6 are also present in the heart [4], but their functions are not completely understood. At least upon aging, overexpression of PP5 leads to cardiac hypertrophy [15]. There is evidence that the aging human heart exhibits similar contractile defects (due to causative alterations in protein expression) as the failing human heart (end-stage heart failure, NYHA IV [16–19]). For instance, one has noted enhanced activity/expression of PP1 and/or PP2A in end-stage human heart failure [20, 21]. Hence, it is conceivable that protein expression of these major serine/threonine phosphatases might be altered in the aging human heart. Others have studied a role of PP in atrial samples in the development of cardiac failure [22]. Moreover, PP1 activity was increased (but not the protein expression of the catalytic subunit of PP1) in atria in a canine model of tachycardia-induced heart failure [22]; in that model, the Ca content of the SR was enhanced in the atria (but reduced in the ventricle); more Ca could be released from the SR and local basal contractility was enhanced [22], underlining the regional differences in cardiac diseases.

Thus, in the present work, we tested the hypothesis that the expressions of the aforementioned catalytic subunits of phosphatases or their ancillary or regulatory proteins are altered in the aging human heart.

A report on the progress of this work has appeared in abstract form [23].

## 2. Materials and Methods

### 2.1. Patients

Our inclusion criteria were male sex and treatment with the *β*-adrenoceptor blocker metoprolol, bisoprolol, or carvedilol. Exclusion criteria were treatment of any thyroid disorder, NYHA class higher than 3, ejection fraction under 35%, and treatment with more than 12 different drugs (one patient). The mean NYHA class was 2.02 ± 0.2, the mean left ventricular ejection fraction was 63.65 ± 8.25%, and the mean CCS was 2.25 ± 0.4. All patients were between 48 and 84 years of age. All patients gave their written informed consent. Patients underwent open heart surgery because of bypass coronary grafting, and small cardiac tissue samples from the right atrial appendage were collected. The study was approved by the ethical committee of the University Hospital in Halle, Germany (hm-bü 04.08.2005), and the study was conducted according to the principles expressed in the Declaration of Helsinki.

### 2.2. Western Blot Analysis

For western blot analysis, tissue homogenates were prepared and 2x strength SDS sample buffer prepared according to Laemmli [24] was added. The samples were solubilized for 10 min at 95°C. Aliquots of protein (100 *µ*g/lane) were loaded per lane, and gels were run using 10% polyacrylamide separating gels. Proteins were electrophoretically transferred to nitrocellulose membranes in 50 mM sodium phosphate buffer (pH 7.4) 180 min at 1.5 A at 4°C as described before [20, 25], using a Hoefer vertical electrophoresis system (Hoefer, Holliston, MA, USA) composed of SE600 standard dual cooled vertical protein electrophoresis unit; TE62 standard transfer tank with cooling chamber. Power Supplies were from Bio-Rad (Bio-Rad Laboratories, Munich, Germany). Then, membranes were treated with TRIS-buffered saline containing 5.0% nonfat dry milk powder, and 0.1% Tween 20 for 60 min at room temperature followed by incubation with primary antibodies overnight at 4°C. Finally, alkaline phosphatase-labeled secondary antibodies were used, and bands were detected using enhanced chemofluorescence (ECF, GE Healthcare Europe, Freiburg, Germany). Fluorescent bands were visualized with a TYPHOON 9410 imager and quantified using the ImageQuaNT software (GE Healthcare Europe, Freiburg, Germany). Following primary antibodies were used: from Santa Cruz, Heidelberg, Germany, diluted at 1 : 1000: anti-PP6 (#sc-130849), anti-PP2C*α*/*β* (#sc-166662), anti-PP2B-B1/2 (#sc-373803), anti-PP2B-A (#sc-9070), anti-PP2A-C*α*/*β* (#sc-14020, 1 : 250), anti-PP2A-B56-*α* (#sc-271311, 1 : 500), anti-PP2A-A*α*/*β* (#sc-74580, 1 : 250), and anti-I2PP2A (#sc-5655, 1 : 1000); from Biomol, Hamburg, Germany: anti-PHAP 1(=I1PP2A, #P3368-02F, 1 : 1000), and anti-PPP1R2 (=I_2_^PP1^, #WA-AP16058b, 1 : 1000); and anti-calsequestrin (#SP5340P, 1 : 1000, Acris Antibodies, Herford, Germany), anti-protein phosphatase inhibitor 1 (#ab40877, 1 : 1000, abcam, Berlin, Germany), anti-PP5 (#611021, 1 : 500, BD Transduction Laboratories, Heidelberg, Germany), and anti-PP1*α* (#2582, Cell Signaling Technology via New England Biolabs, Frankfurt/Main, Germany). Secondary antibodies (anti-goat IgG, anti-mouse polyvalent immunoglobulins, and anti-rabbit IgG) were diluted 1 : 1000 or 1 : 500 (all from Sigma-Aldrich, Hamburg, Germany) and incubated for 2 hours at room temperature. Using these antibodies, we first studied linearity of loaded protein versus antibody detection in typical human cardiac samples. We noted the linear range from 25 to 200 *µ*g protein per lane (data not shown) for all antibodies studied. In all subsequent experiments, we used those amounts of antigen (that is, human atrial homogenates) that were within these linear ranges. On each gel, a reference sample was run which was used to compare between gel runs.

### 2.3. Statistics

Data are presented as means ± SEM. Comparisons between groups were evaluated using one-way ANOVA followed by Bonferroni's test for multiple-group comparisons. The data were tested to be normally distributed. For Table 1, we used a multivariate analysis. For calculation of correlations and analyses of variance, we used the software Prism 5 (GraphPad Software, La Jolla, CA, USA). A value of *p* < 0.05 was considered statistically significant.

## 3. Results

**Figure 1:**
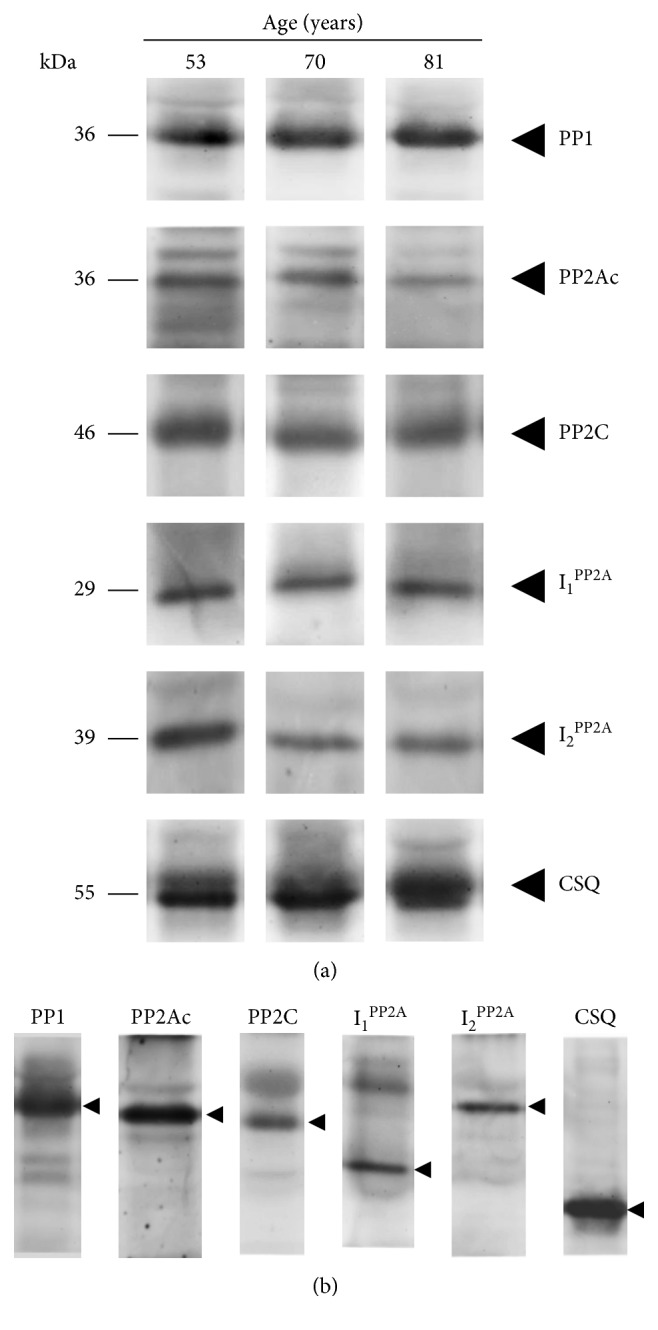
Representative western blots for PP1, PP2Ac, PP2C, I_1_^PP2A^, and I_2_^PP2A^ of cardiac homogenates from patients of 53, 70, and 81 years of age (a). Exemplary full lanes of western blots are also depicted (b); as loading control calsequestrin (CSQ) was studied. On the top of each lane, the age of the patient is given.

**Figure 2:**
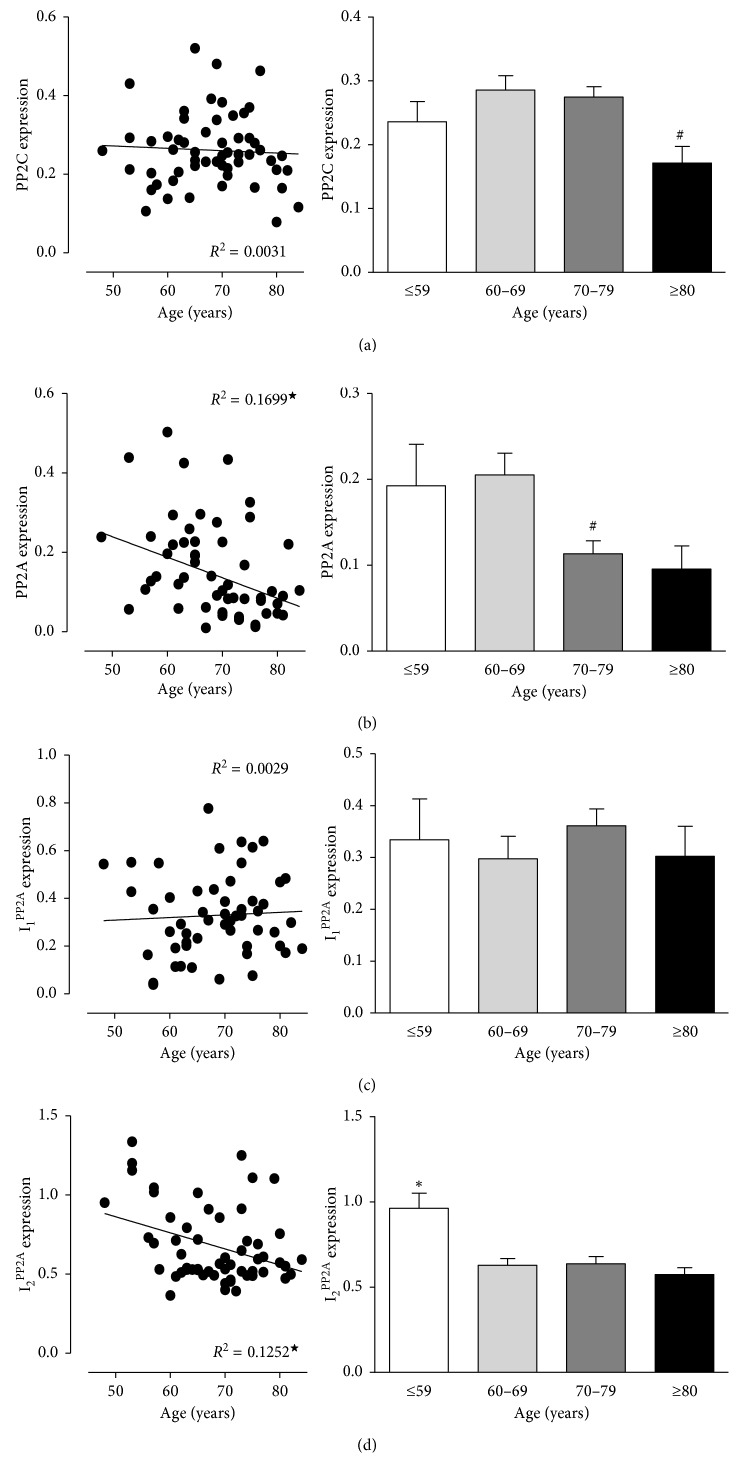
Correlation of PP2C (a), PP2Ac (c), I_1_^PP2A^ (e), and I_2_^PP2A^ (g) expression with age. Ordinates are expression data normalized to CSQ in arbitrary phosphoimager units versus age (abscissae). (b), (d), (f), and (h) Expression data are combined to four age groups and are given as means ± SEM; *n*=6 − 24 (compare with Table 1). ^★^ indicates a significant correlation, ^*∗*^*p* < 0.05 vs. all other age groups, and ^*#*^*p* < 0.05 vs. age group 60–69.

Antibody specificity was first validated using western blotting. Homogenates were prepared from frozen human atrial samples and subjected to western blotting. Linearity of the assay in a range of 25 to 200 *µ*g protein was established initially for all proteins subsequently studied (data not shown) and finally, western blotting experiments were performed using 100 *µ*g proteins per lane. Exemplary full lanes of western blots are also depicted in Figure 1(b), and typical western blots for proteins of interest in typical age groups are presented in Figure 1(a). Similar experiments were done with all other proteins of interest, namely, the catalytic subunit of PP1, the catalytic subunit PP2Ac, the regulatory subunits A and B56*α* of PP2A, the inhibitory subunits I_1_^PP2A^ and I_2_^PP2A^ of PP2A, the inhibitory subunits I_1_^PP1^ and I_2_^PP1^ of PP1, and the catalytic and regulatory subunits of calcineurin (PP2B), PP5, and PP6. Calsequestrin (CSQ) was used as a loading control as we published before [10, 15]. All antibodies showed specific labeling of proteins with the expected molecular weights (Figure 1). Of note, there was a decreased expression of PP2Ac on protein level in aging (see lanes in the second row from the top in Figure 1(a)) and a decrease of I_2_^PP2A^ upon aging (see lanes in the second row from bottom in Figure 1(a)). This initial observation was corroborated by studying more samples and performing a statistical analysis (see Figure 2).

The patient characteristics can be seen in Table 2. The medications are typical for angina pectoris patients. All additional drugs are typical for this age group in our hospital. The percentage of drug use can also be seen.

Figure 2 shows no linear correlation between age and expression of PP2C (Figure 2(a)). However, a subgroup analysis depicts decreased expression of PP2C in the oldest group (≥80) vs. 60–69. Figure 2 indicates a significant negative correlation between PP2Ac (Figure 2(b)) as well as I_2_^PP2A^ (Figure 2(d)) expression and age. In line with that, patients older than 60 years of age (I_2_^PP2A^) or 70 years of age (PP2Ac) exhibited reduced expression of PP2Ac and I_2_^PP2A^, respectively. No linear correlation between age and expression of I_1_^PP2A^ was noted (Figure 2(c)). Likewise, a subgroup analysis revealed no altered expression of I_1_^PP2A^ (Figure 2(c)) between the groups.

Furthermore, we did not observe any significant correlation (Table 2, multivariate analysis) between age and the other proteins which were studied in this work, namely, the catalytic subunit of PP1, the regulatory A-subunit of PP2A, the regulatory B56*α*-subunit of PP2A, I_1_^PP1^, and I_2_^PP1^, and the catalytic and regulatory subunits of calcineurin, PP5, and PP6 (*p* > 0.05) and subgroup of decennial age group analysis did not reveal age-dependent significant difference with regard to these parameters (data not shown).

## 4. Discussion

Atrial tissue from patients undergoing bypass surgery due to coronary heart disease was studied in the present work. All patients included in this study were on *β*-adrenoceptor blocker therapy. *β*-Adrenoceptor blockers can alter many of the biochemical parameters studied here [26]. Several studies on gene expression during aging in animal models [27] and in human tissue also using gene expression arrays [28] have been published. In humans, 162 candidate gene products correlating with heart failure were identified. However, only mRNA for methionine tRNA synthase correlated with age [28]. In nonfailing human hearts, only two transcripts correlated with age, for instance, the abundance of metallothionein 1L increased with age [28]. However, these data were obtained in ventricular tissue and on mRNA levels, whereas we studied atrial tissue and protein expression.

In the past, we and others have described an increased expression of PP1 and/or PP2A in animal models of heart failure [20, 21, 26, 29, 30]. Moreover, we detected increased activity of PP1 in the SR of failing human hearts [20]. These data are supported by animal studies with overexpression and knockout mice. For instance, overexpression of the catalytic subunits of PP1, PP2A, or PP5 can lead to hypertrophy or heart failure in transgenic mice [8, 10, 15, 31]. The mechanism of hypertrophy is thought to involve dephosphorylation of key regulatory proteins in a specific way by phosphatases [5]. One of the typical substrates for PP2A and PP1 and a classical regulator of cardiac contractility is phospholamban [32]. Phosphatase 1- or 2-induced hypertrophy and/or failure can be attenuated or abolished by co-overexpression of I_1_^PP1^ or I_2_^PP1^ in double transgenic animals or transfection of myocytes from failing hearts with a virus coding for the appropriate inhibitor [7,33–39]. Expression and function of I_1_^PP1^ due to regulation by micro-RNA765 might be reduced in heart failure [40–42].

Interestingly, treatment of rat hearts with isoproterenol, a classical model of cardiac hypertrophy, increased phosphatase activity (PP1 and PP2A), whereas coadministration of the *β*-adrenoceptor blocker propranolol not only inhibited cardiac hypertrophy but also normalized the activity of phosphatases [26]. These data indicate that part of the beneficial effects of treating patients with heart failure with *β*-blockers may result from reducing phosphatase activity. Furthermore, these data imply that *β*-adrenoceptor blocker treatment may alter phosphatase expression, and therefore we included only patients that were on *β*-adrenoceptor blocker treatment in order to rule out this treatment as a confounding factor. In contrast, to the findings with heart failure and our initial assumption, we noted no alterations in these well-defined proteins that are implied in heart failure, namely, the catalytic subunit of PP1 and the classical inhibitors of PP1 were found unaltered. Furthermore, there is good evidence that the activity of calcineurin (=PP2B) is increased in human heart failure; PP2B has been overexpressed in animal models, leading to heart failure and hypertrophy [43]. However, in our study samples, protein expression of PP2B (A or B subunit) was unchanged. The picture was somewhat different for PP2A. However, the B and A subunits of PP2A and the I_1_^PP2A^ were unchanged, and the expression of the catalytic subunit PP2Ac and I_2_^PP2A^ declines with age (Figure 2). This decline is expected to lead to changed PP2A activity. Scarce data are available on the cardiac regulation of I_2_^PP2A^ expression. However, in hearts from rats treated with isoproterenol to induce hypertrophy, an increase in the mRNA coding for I_2_^PP2A^ (SET) was noted [44]. Hence, we suggest that decline in PP2A (but for different biochemical reasons) is a common feature in cardiac aging and heart failure. Another novel finding of this work is the reduced expression of PP2C in the oldest group of patients. Genetic knock down of PP2C in zebrafish led to heart failure [45]. Moreover, PP2C expression is elevated in hearts from obese rats, and thus PP2C has been linked to lipotoxic cardiomyopathy [46]. In the meantime, we have successfully generated mice with cardiac specific overexpression of PP2C [47]. In these animals, we plan to test whether their hearts are functionally altered in aging. However, as PP2C was found in cardiac mitochondria [4, 45], mitochondrial function and gene expression is altered in heart failure [48]; a role of PP2C is cardiac aging is not unreasonable to assume. Limited data are available on the expression and/or activity of phosphatases in the heart of experimental animals. Several years ago, we have shown that the expression of the mRNA and the protein levels as well as the activity of PP1 and PP2A greatly decline in adult versus neonatal rat hearts [49]. However, neonatal human hearts for these studies were not available to us. Nevertheless, those data [49] clearly have shown that PP1 and PP2A can be regulated upon development and aging in the mammalian heart. PP6 shows 57% sequence homology to the catalytic subunit of PP2A and is highly expressed in human heart and may be involved in cell cycle regulation [50] but was unchanged in the present study.

### 4.1. Study Limitations

One drawback of the present study can be seen in the fact that we have only studied diseased myocardium. However, nonfailing myocardium, more specifically atria from nondiseased hearts are not available to us as present in our institution, and such data, perhaps obtained via noninvasive methods, are awaited with interest. Moreover, due to lack of tissue, we have not been able to study the ventricular myocardium. In the past, we have described that the distribution of phosphatases in the human heart is different between atrium and ventricle [51]. In detail, we noted that the mRNA of the isoforms of PP1 was higher in the right ventricle than in the right atrium. However, the protein expression as studied by western blots was not different between these tissues, underscoring the value of measuring protein expression of phosphatases. In contrast, mRNA of isoforms of PP2A as well as protein was higher in right ventricles compared to human right atria [51]. Another serious limitation of the present study was that all patients obtained several drugs and we cannot exclude that some of the changes that are present in the aging myocardium were obscured by drug effects. In addition, we excluded severely ill patients (NYHA IV) because that was expected to bias our result as many gene alterations are known in end-stage heart failure. Indeed, EF fractions were in the normal range arguing for the absence of (systolic) heart failure in the study patients. Moreover, we are currently lacking tissue to study whether (as predicted) the PP2A enzyme activity really increases upon aging in human hearts. In pathological aging, at least in brain a related but functionally opposite mechanism has been described: SET (=I_2_^PP2A^) is released from the nucleus to the cytosol and inhibits PP2A activity; this leads to hyperphosphorylation of tau which may manifest as Morbus Alzheimer [52]. Reduced PP1 activity as a result of I_1_ overexpression has been studied in aging mice. Here, somewhat conflicting results are obtained: [7] reported that I_1_ overexpression led to cardiac hypertrophy with age, whereas others noted no cardiac hypertrophy (though increased phosphorylation of cardiac regulatory proteins) with a lower level of I_1_ overexpression in transgenic mice [53, 54]. Nevertheless, these data are constituent with a role of PP in the aging heart and might underscore that the extent of PP alteration through modulation of endogenous PP inhibitors is relevant.

Furthermore, one can question why we measured the expression of our proteins of interest (PP) with regard to cardiac calsequestrin (CSQ-2). Calsequestrin has the advantage of being a protein mainly if not exclusively expressed in cardiomyocytes. The group of Knollmann and we ourselves have generated CSQ KO mice. Indeed, in these mice, we detected no signals in the atrium of CSQ2 KO mice [55], proving that the antibody we used also in the present study solely detects CSQ-2. Herraiz-Marinez [56] noted the ratio of CSQ-2/GAPDH to decline with aging, and thus one might ask whether we should not use GAPDH instead of CSQ-2 as a reference in our samples. However, GAPDH is a well-accepted housekeeping protein; it is present in cardiomyocytes but also abundantly present in nonmyocytes. Hence, in the course of aging or the underlying coronary heart disease (which led to surgery in our patients), a proliferation of nonmyocytes might have occurred (for instance, an increase in the number of fibrocytes). Hence, we think for the time being that to measure against CSQ-2 is an alternative valid approach if one wants to refer to expression with regard to cardiomyocytes.

### 4.2. Conclusions

The main new finding is that phosphatases are part of the aging process in the human heart. More detailed studies are necessary to clearly define whether they might be targets for drug therapy in aging. For instance, we can speculate that drugs that inhibit selectively PP2A might be beneficial. Such a drug is in principle available with okadaic acid: at 10 nM, it inhibits only PP2A not PP1 [4]. Regrettably, its action is not restricted to the myocardium, and we have shown that, by inhibition of smooth muscle phosphatases, it leads to vasoconstriction in isolated human coronary arteries which would worsen cardiac function [57]. We speculate here that the search for cardiac muscle specific inhibitors of PP2A might be reasonable for the pharmacological treatment of the aging human heart.

## Figures and Tables

**Table 1:** Protein expression data of decennial patient groups.

	≤59	60–69	70–79	≥80
Catalytic subunit of PP1 (phosphoimager-units)	1.35 ± 0.29	0.99 ± 0.056	1.26 ± 0.16	1.08 ± 0.15
Catalytic subunit of PP2A (phosphoimager-units ∗ 10^−1^)	1.93 ± 0.48	2.05 ± 0.27	1.13 ± 0.24	0.96 ± 0.27
Regulatory A subunit of PP2A (phosphoimager-units)	1.47 ± 0.20	1.24 ± 0.16	1.52 ± 0.13	0.81 ± 0.20
Regulatory B56*α*-subunit of PP2A (phosphoimager-units ∗ 10^−1^)	4.26 ± 0.73	3.69 ± 0.43	2.88 ± 0.33	2.62 ± 0.76
I1PP1 (phosphoimager-units ∗ 10^−1^)	7.85 ± 1.41	9.29 ± 0.91	7.50 ± 0.77	9.30 ± 1.45
I2PP1 (phosphoimager-units ∗ 10^−2^)	12.80 ± 2.50	12.76 ± 1.49	9.29 ± 1.35	4.74 ± 1.76
A subunit of PP2B (phosphoimager-units)	1.55 ± 0.31	1.04 ± 0.13	1.02 ± 0.10	1.13 ± 0.28
B subunit of PP2B (phosphoimager-units ∗ 10^−1^)	2.63 ± 0.46	2.46 ± 0.36	2.34 ± 0.23	1.75 ± 0.51
PP6 (phosphoimager-units ∗ 10^−2^)	4.67 ± 0.64	6.90 ± 0.99	6.76 ± 0.88	4.71 ± 0.85
PP5 (phosphoimager-units ∗ 10^−1^)	6.27 ± 1.13	5.20 ± 0.65	5.48 ± 0.51	5.52 ± 0.61

**Table 2:** Patient characteristics.

Age groups (years)	*N*	*β*-Blockers	Statins	ASS	ACE inhibitors	AT_1_ antagonists
≤59	9	9	7 (78)	6 (66)	6 (66)	1 (11)
60–69	21	21	14 (66)	12 (57)	11 (52)	2 (95)
70–79	24	24	19 (79)	14 (58)	18 (75)	2 (83)
≥80	6	6	4 (66)	4 (67)	5 (83)	0 (0)
Total	60	60	44 (73)	36 (60)	40 (67)	5 (83)

All patients received *β*-adrenoceptor blockers as required by our inclusion criteria. Percentage of patients receiving per row the other drugs are given in percentage in brackets in order to facilitate comparison.

## Data Availability

The data used to support the findings of this study are available from the corresponding author upon request.
